# Insulin-Like Growth Factor (IGF) System in Liver Diseases

**DOI:** 10.3390/ijms19051308

**Published:** 2018-04-27

**Authors:** Agnieszka Adamek, Aldona Kasprzak

**Affiliations:** 1 Department of Infectious Diseases, Hepatology and Acquired Immunodeficiencies, Poznan University of Medical Sciences, 3 Szwajcarska Str., 61-285 Poznań, Poland; 2 Department of Histology and Embryology, Poznan University of Medical Sciences, 6 Swiecicki Str., 60-781 Poznań, Poland; akasprza@ump.edu.pl

**Keywords:** insulin-like growth factor system, liver, nonalcoholic fatty liver disease, cirrhosis, hepatocellular carcinoma

## Abstract

Hepatocyte differentiation, proliferation, and apoptosis are affected by growth factors produced in liver. Insulin-like growth factor 1 and 2 (IGF1 and IGF2) act in response to growth hormone (GH). Other IGF family components include at least six binding proteins (IGFBP1 to 6), manifested by both IGFs develop due to interaction through the type 1 receptor (IGF1R). The data based on animal models and/or in vitro studies suggest the role of IGF system components in cellular aspects of hepatocarcinogenesis (cell cycle progression, uncontrolled proliferation, cell survival, migration, inhibition of apoptosis, protein synthesis and cell growth), and show that systemic IGF1 administration can reduce fibrosis and ameliorate general liver function. In epidemiologic and clinicopathological studies on chronic liver disease (CLD), lowered serum levels, decreased tissue expression of IGF1, elevated production of IGF1R and variable IGF2 expression has been noted, from the start of preneoplastic alterations up to the developed hepatocellular carcinoma (HCC) stage. These changes result in well-known clinical symptoms of IGF1 deficiency. This review summarized the current data of the complex role of IGF system components in the most common CLD (nonalcoholic fatty liver disease, cirrhosis, and hepatocellular carcinoma). Better recognition and understanding of this system can contribute to discovery of new and improved versions of current preventive and therapeutic actions in CLD.

## 1. Introduction

The liver is a key organ in insulin-mediated regulation of metabolism. In addition to its key role in whole-body, glucose and lipid homeostasis, this organ is also a major site of the synthesis of plasma proteins and endocrine factors such as insulin-like growth factor 1 and 2 (IGF1 and IGF2), as well as their binding proteins (IGFBPs) and thereby influences whole-body metabolism and growth [1]. The circulating IGFs do not represent the true level of tissue secretion, though they may reflect organ development to some extent [2]. Moreover the role of the liver in the secretion of IGF1 and IGFBPs, is not only under endocrine and nutritional control, but also under autocrine and paracrine control [3]. The IGF regulatory system in each organ is tissue-specific, but all share similar components including specific ligands (both IGFs), IGFBPs (no. 1–8), IGF receptors (type I and II), and IGFBP-specific proteases [4,5].

IGF1 and IGF2 were named insulin-like growth factors because of their structural homology with insulin (~50% identical sequence) and similar metabolic actions. They are functionally related to insulin but have a much higher growth-promoting activity [6,7]. Quantitatively, IGF2 is the predominant circulating IGF, present in adults at a concentration up to ~700 ng/mL of blood, three times that of IGF1 [8,9].

IGFs are a part of the insulin-related family together with relaxin, with genes encoding members of that family located on distinct genomic fragments (chromosome 2 and 11) [10]. In mammals, liver-derived endocrine IGF1 mediates the growth promoting effects of growth hormone (GH) during postnatal life, whereas IGF2 stimulates placental and fetal growth, and is less GH dependent than IGF1 [8,9]. However, results of in vivo and in vitro studies, describing GH as a physiological regulator of IGF2 gene expression in humans in a promoter-specific way are also present [11]. 

The IGF1 gene is well-conserved among 25 different mammalian species representing 15 different orders and ranging over ~180 million years of evolutionary diversification [12]. IGF1 plays a central role in pre- and postnatal growth in humans and other mammals, as a key mediator of the GH actions, while also being involved in the control of intermediary metabolism, tissue repair and disease pathogenesis throughout life [13,14,15]. Both IGFs are central hormones involved in metabolic signaling, affecting glucose uptake, lipogenesis, glycogen storage, and suppression of protein degradation [16,17]. IGF1 is necessary for normal insulin sensitivity, and impairment of IGF1 synthesis results in a worsening state of insulin resistance [18]. 

IGFs in biological fluids are associated with IGF binding proteins (IGFBPs), which are the principal regulators of IGFs’ bioactivity and activity in metabolic signaling pathways. Moreover, these proteins extend the half-life of IGFs in the bloodstream, store IGFs in specific tissue compartments, inhibit activity of IGFs by lowering accessibility of their receptors, and protect them from proteolytic degradation [5,19,20,21]. 

In blood ~90% of IGF1 is associated with IGFBP3, circulating as a 150 kDa complex that consists of IGF1, IGFBP3, and an acid-labile subunit (ALS) [22]. Free IGF1 has a half-life of ~8 min in serum. This can be increased to ~30 min if bound to IGFBP3 and up to ~15h in the ternary complex with IGFBP3 and ALS [16]. 

IGFs affect cells through specifically binding three various surface receptors, type-I IGF receptor (IGF1R), type-II IGF receptor (IGF2R), insulin receptor (IR) and hybrid receptors (IGF1R/IR) [23,24]. Most activities of both IGFs are mediated by a type I receptor, with these interactions regulated by high-affinity IGFBPs, which can inhibit or enhance the action of IGFs and IGF low-affinity IGFBP-related proteins (IGFBPrP1-10). In addition, IGFBPs’ activity is also regulated by IGFBP proteases [14,19,24,25].

The main functions of IGF system in liver physiology include the role of the system in organ development, growth and regeneration. At the cellular level, both IGFs (endocrine and autocrine/paracrine), as well as their receptors (IGF1R, IGF2R), regulate cell cycle progression, proliferation and hepatocytes differentiation [25]. Generally, the components of IGF system are hepatoprotective, playing a very important role in hormonal and metabolic effects [1,17,18,20]. The topic of whole IGF system in chronic liver diseases (CLD) that lead to primary liver cancer, is of particular interest, since a better understanding of the role of the IGF system in cancer could promote novel approaches to diagnosis and therapeutic strategies for this disease [15,25]. 

This review summarized the current data of the complex role of IGF system components in the most common CLD (nonalcoholic fatty liver disease, cirrhosis, and hepatocellular carcinoma). Better recognition and understanding of this system can contribute to discovery of new, as well as improvement of current preventive and therapeutic actions in CLD.

## 2. Role of IGF System in the Pathogenesis of Chronic Liver Disease (CLD)

Among several physiological functions of IGF system components is their role in the pathogenesis of CLD, which can lead to hepatocellular carcinoma (HCC) and end-stage liver disease. Diseases, which are classified as CLDs, include: compensated cirrhosis of any etiology, chronic hepatitis, alcoholic liver disease (ALD), nonalcoholic fatty liver disease (NAFLD), cholestatic liver disease, and metabolic liver disease [26,27]. 

This review will include studies of the role of IGF system in NAFLD, cirrhosis and HCC, as well as liver disease associated conditions.

Nonalcoholic fatty liver disease, defined as abnormal accumulation of triglycerides in the liver (i.e., >5%) in the absence of significant alcohol intake, is recognized as the most common cause and form of CLD, and is estimated to affect 30% of adults and 10% of children in the United States [28], with its worldwide prevalence continuously increasing, in correlation with the growing obesity epidemic [26,29,30]. This form of CLD encompasses a spectrum of histologic findings, including uncomplicated steatosis, steatosis with inflammation and steatohepatitis (nonalcoholic steatohepatitis, NASH). The latter can advance to cirrhosis and HCC [26]. It is also recognized that NAFLD is the hepatic manifestation of metabolic syndrome [31]. The pathogenesis of NAFLD includes metabolic stress to the liver, associated with insulin resistance with downstream cell stress from reactive oxygen species, and unfolded protein response, with activation of inflammatory and fibrotic pathways [30]. Reduction of IGF1 expression caused by inflammatory cytokines is associated with the development of NAFLD, as well with the degree of NAFLD advancement [32]. Patients with adult GH deficiency show increased prevalence of NAFLD/NASH, with GH replacement therapy shown to improve these conditions [33].

Liver cirrhosis is the end stage of many acute and CLD, affecting innumerable patients worldwide. There are many reasons for liver cirrhosis, including HBV and HCV infections, alcohol abuse and cholangiopathies. It is characterized by fibrotic replacement of liver tissue, necrosis and regeneration nodules that lead to loss of functional liver mass [34,35]. 

HCC is the second cause of death due to malignancy in the world, following lung cancer. Most cases of HCC are related to liver diseases, including liver cirrhosis due to HCV or HBV infections [36]. 

As the IGF system plays an important role in the development of the most common CLDs, the use of animal models (including transgenic animal technology) has been preferred for experiments concerning liver diseases, in vitro studies, and collection of epidemiologic and clinicopathological data. Current epidemiologic studies confirm a correlation between the serum levels of IGF system components and the extent of hepatocellular function (see below).

## 3. Evidence from Animal Models

### 3.1. Role in Nonalcoholic Fatty Liver Disease (NAFLD)

Concerning the role of the GH/IGF1 system in the pathogenesis of NAFLD/NASH, it has been reported that decrease in the levels of system components is closely associated with the progression of NAFLD [33]. Low IGF1, and IGF1/IGFBP3 ratio may be associated with advanced liver fibrosis, while low levels of GH might have a role in hepatic steatosis in NAFLD [33,37]. GH may be involved in the mechanism of hepatocyte triglyceride secretion [37].

To date, several studies on animal models demonstrated a potential role of the IGF system in pathogenesis of NAFLD/NASH. Rats with the history of prenatal administration of dexamethasone present NAFLD in adulthood, together with decreased IGF1 plasma concentrations [38]. There are reports on metabolic consequences of prenatal androgenization, that include fatty liver and perturbed insulin signaling in young adult sheep, as well as intrinsic alterations in hepatic gene expression that may contribute to the overall metabolic phenotype observed in these animals [39]. In adult sheep, maternal testosterone propionate exposure resulted in increased insulin secretion to glucose load, as well as histological presence of fatty liver independent of central obesity, with upregulation of IGF1 expression [39]. In another study on spontaneous dwarf rats (SDR), (a GH-deficient rat model) with liver steatosis and fibrosis were evident. Significant decrease of serum AST and ALT levels and liver triglyceride were observed, in comparison to control, after treatment with GH and IGF1. An interesting observation was made in livers of SDR. The mitochondrial morphology of SDR hepatocytes was impaired, with the area significantly decreased and oxidative stress enhanced. These changes were also improved after GH and IGF1 administration, suggesting that GH-independent, IGF1 action plays an essential role in the liver [40]. Recently, the same group of researchers investigated the effect of IGF1 on NASH and cirrhotic mice models and demonstrated that IGF1 ameliorated steatosis, inflammation, and fibrosis. In the study of NASH model with methionine-choline-deficient diet-fed, *db*/*db* mice (MCD-*db*/*db*), IGF1 administration ameliorated histological changes. It worked similarly in mice of a dimethyl nitrosamine (DMN)-induced cirrhotic model, also leading to a biochemical improvement. The latter model has also confirmed that IGF1 in particular, directly inactivates HSCs, limits fibrosis in a p53-dependent manner, and may be applied to treat NASH and cirrhosis [41]. Other experimental studies on rabbits with food restriction showed the development of NAFLD in all cases. At the end of experiment (at necropsy), IGF1 was moderately higher in NAFLD than in control groups [42]. In another study, two groups of mice—one on a choline-deficient L-amino-acid-defined-diet (CDAA), and the other on CDAA supplemented with carbon tetrachloride (CCl4)—developed extensive steatosis and fibrosis (NAFLD/NASH) after 1–3 months. Hepatocellular carcinoma was recognized in 100% of CDAA+CCl4 treated mice and 40% of CDAA mice after 9 months of treatment. In both groups, IGF2 mRNA started to present increased levels at 3 months, with highest expression observed when the cancer appeared [43]. According to the results of this research, CDAA model promoted the development of HCC from NAFLD/NASH in the presence of insulin resistance, but in the absence of cirrhosis [43]. Recent studies on the mice model have demonstrated that transient hepatic IGF2 overexpression can induce a fatty liver, characterized by increased free cholesterol and phospholipids, leading to accumulation of lipid droplets. The study concluded that IGF2 can play a causal role in steatosis initiation [44].

In summary, the animal models help to understand the role of IGF system in the development of NAFLD/NASH and hepatocarcinogenesis in NAFLD. It was proven that GH-deficiency promotes the development of NAFLD/NASH, with IGF1 playing an essential role in its prevention. Using animal models, it was demonstrated that GH/IGF1 treatment shows different hepatoprotective, antioxidant and mitochondrial-protective effects, suggesting potential clinical applications of both of these hormones. Furthermore, the animal models allow to confirm that, particularly IGF1, directly inactivates HSCs, limits fibrosis in a p53-dependent manner and may be applied to treat NASH. IGF2, meanwhile, can be a key factor in the development of steatosis that accompanies NAFLD/NASH.

### 3.2. Role in Liver Cirrhosis

The availability of animal models for experimental liver cirrhosis (carbon tetrachloride, thioacetamide, bile duct ligation, d-galactosamine, etc.) helped to better clarify the role of IGF1 in this pathology. It was proven, by numerous studies, that treatment with IGF1 can be beneficial in liver cirrhosis [35]. A recombinant simian virus 40 vector (rSV40), encoding IGF1 (rSVIGF1), was first evaluated in mice injected with rSV40 encoding luciferase, which showed long-term hepatic expression of the transgene. Then, rSVIGF1 therapeutic efficacy was studied in rats, in which liver cirrhosis was induced by carbon tetrachloride (CCl_4_) inhalation and showed that the hepatic levels of IGF1 and IGFBP3 were higher in rSVIGF1-treated rats than in control cirrhotic animals. Hence, it was concluded that rSV40-mediated, sustained expression of IGF1 in the liver slowed cirrhosis progression [45]. In other experimental models of CCl_4_-induced cirrhosis in animals, administration of rhIGF1 improved food intake [46], carbohydrates and amino acids intestinal absorption [47,48], muscle mass [46,47,48], osteopenia [49], testicular atrophy [50], and normalized testicular transferrin expression, as well as reduced serum levels of LH [51]. IGF1 therapy has also been proved to revert insulin resistance, reduce cholesterol and triglycerides levels, and significantly increase FFA concentrations [52]. It has also been proven, that systemic IGF1 administration decreased collagen levels in liver and histological fibrosis score, ameliorated liver function and reduced fibrosis [53,54,55]. Additionally, it has been observed to upregulate hepatocyte growth factor (HGF) and matrix metalloproteinases (MMPs), downregulate tissue inhibitor of metalloproteinase 1 (TIMP-1) [56], reduce portal pressure, bacterial translocation and endotoxemia [55]. Additionally, an increase in albumin, total protein and coagulation factor (II, VII, X) levels, as well as decrease in liver lipid peroxidation product, was observed [53]. The study on rat model of insulin-like growth factor binding protein-related protein 1 (IGFBPrP1)-overexpressing livers, has confirmed the results of in vitro studies (see below), stating that cellular IGFBPrP1 was upregulated in fibrotic and cirrhotic liver specimens. IGFBPrP1, known as Mac25 or IGFBP7, plays the role of a tumor suppressor. In studies, the protein induced hepatic stellate cells (HSCs) activation and extracellular matrix (ECM) production. Additionally, the authors showed that the overexpression of IGFBPrP1 induced hepatocyte apoptosis and HSC activation in a Smad-dependent manner [57]. Recently, it was investigated that IGF1 significantly ameliorated fibrosis in cirrhotic model of dimethyl nitrosamine-treated mice. IGF1R was strongly expressed in HSCs, with IGF1-induced cellular senescence in HSCs, in vitro and in vivo conditions [41]. 

Summarizing, through studies on animal models, it was concluded that GH/IGF1 treatment generally improves liver function and reduces fibrosis. The animal models have also allowed to confirm that, especially IGF1, directly inactivates HSCs, limits fibrosis in a p53-dependent manner, and may be applied to treat liver cirrhosis [41]. Similar to NAFLD/NASH, IGF1 treatment shows different positive hepatoprotective, antifibrogenic, antioxidant and mitochondrial-protective effects in cirrhotic livers [35]. The studies on animal models have also greatly contributed to our understanding of the role of less known IGF system components, e.g. IGFBPrP1 (IGFBP7), in the pathogenesis of liver cirrhosis.

### 3.3. Role in Hepatocellular Carcinoma (HCC)

Liver carcinogenesis develops through multiple genetic alterations and protein expressions. The animal models revealed, that one of the most important factors in this process is IGF2. In transgenic mice, in which HCC was the most common tumor, IGF2 levels were 20-fold higher than in healthy control mice [58]. In early experimental HCC in rats, IGF2 was expressed in the cytoplasm of both sinusoidal cells in precancerous cirrhotic liver tissue and malignant hepatocytes. This expression was also present in the rough endoplasmic reticulum and mitochondria of malignant hepatocytes. IGF2 mRNA levels were higher in liver tumor tissue than in that of normal rats [59]. In diethyl nitrosamine (DEN)-treated and phenobarbital promoted hepatocarcinogenesis in rats, high expression of IGF2 mRNA was present. The expression of IGF2 was found to be predominant in the HCC, but seen also at the peripheral cells of spongiosis hepatis, which are believed to be the precursors of Ito cell carcinoma [60]. In hepatoma models, induced by 2-fluorenylacetamide (2-FAA) in rats, progressively increasing hepatic IGF2 levels during HCC development were observed [61]. In streptozotocin-induced diabetic rats, hepatocellular adenomas and HCC developed after a sequence of characteristic preneoplastic hepatic foci. Some of these HCCs reached even a 100-fold overexpression of IGF2. HCC tissue consistently showed an increased IGF1R expression, rendering these tissues susceptible to the mitogenic effects of IGF. The altered gene expression in glycogen-storing preneoplastic hepatic foci, especially the upregulation of IGF1 and IGFBP4, with the downregulation of IGFBP1, resemble the insulin-dependent regulation of these components in normal rat hepatocytes [62]. Other studies on *N*-nitroso morpholine-induced rat hepatocarcinogenesis confirmed the increase in IGF1R levels in liver, which may lead to increased cell proliferation in initial phases of hepatocarcinogenesis [63]. On the other, 2-FAA-induced, rat hepatoma model, dynamic expression and alterations in IGF1R and IGF1R mRNA, were observed in different stages of malignant transformation [64].

The data above indicates that both IGFs and IGF1R participate in hepatic carcinogenesis. Firstly by promotion of hepatocyte proliferation through the paracrine mechanism in the precancerous stage, and secondly in malignant cell proliferation, induced by the autocrine mechanism [58,59,60,61]. 

## 4. Evidence from In Vitro Studies

### 4.1. Role in Nonalcoholic Fatty Liver Disease (NAFLD)

It has been shown that IGF1 has a direct an anti-inflammatory effect on hepatic cells [32]. HepG2 cells were incubated with IL-6, in the presence or absence of IGF1. IGF1, at physiologic or supraphysiologic concentrations, reverted the effects of IL-6. Additionally, a downregulation of C-reactive protein (CRP) and fibrinogen mRNA transcripts, and an upregulation of albumin mRNA transcripts were observed [32]. Zhang et al. demonstrated a significantly decreased expression of IGF1 mRNA, as well as IGFBP3 and IGF1 proteins in nonalcoholic fatty steatosis, in immortalized human hepatocyte models, as compared with the control group [65]. Other studies detected insulin and IGF1 receptors in cultured rat HSCs, stimulating HSC proliferation in dose-dependent manner. IGF1 increased type I collagen gene expression and its accumulation in HSCs, which promoted liver fibrosis in vivo [66]. Enhanced IGF1 signaling (silencing IGF1R expression by addition of IGF1 or small interference RNA) inhibits oxidative-stress induced apoptosis in human umbilical vein endothelial cells (HUVECs), by reducing mitochondrial dysfunction [67]. In vitro exposure of HuH7 cells to high concentrations of free fatty acids (FFA), resulted in fat congestion, which favored inflammatory and fibrogenic response, similar to the one observed in patients with NAFLD and NASH. Intracellular lipid accumulation was associated with an increment in IGF2 gene expression [68]. 

In summary, the in vitro experiments allowed to elucidate the clinical significance of both IGFs in NAFLD and its relationship with inflammatory biomarkers, fibrosis, and lipid accumulation. The studies on cultured cells demonstrated that IGF1 can directly modulate the expression of acute-phase reactants, decreasing CRP and fibrinogen levels and upregulating albumin expression. Some of the results will provide the experimental basis for further, clinical studies of the mechanisms of nonalcoholic fatty liver disease.

### 4.2. Role in Liver Cirrhosis

Changes in the IGF system are well documented in liver cirrhosis, resulting in a progressively impaired hepatocellular function [34].

In vitro models of study are concentrated primarily on pathogenesis of hepatic fibrosis (fibrogenesis) and liver regeneration. The hepatic stellate cells play a very important role in processes of liver regeneration (regulation of EMC composition) and fibrosis [69]. Oxidative stress, cytokines and lipopolysaccharides can provoke activation of HSCs to myofibroblast phenotype [70]. HSCs are the target of IGF1. Early cultured, rat HSCs are more susceptible to IGF1 mitogenic action than culture-activated HSCs. Higher expression of IGF1R and lower of IGFBP3 and -4, were observed in early cultured HSCs. That data suggests a role of the IGF system components in the initiation, rather than the maintenance of HSCs proliferation during fibrogenesis [71,72]. Other authors also showed IGF1-stimulated HSCs proliferation in a dose-dependent fashion (four to five times more potent than insulin). Likewise, IGF1 increased type I collagen gene expression and accumulation in HSC culture media, through a PI3-K and extracellular-regulated kinase (ERK)-dependent mechanisms [66]. HSCs produce HGF in the normal liver. During fibrogenesis, they produce TGFβ1, which plays the role of an inhibitor of hepatocyte proliferation. Addition of IGF1 to rat HSC cultures resulted in time- and dose-dependent increase of HGF, but without an effect on TGFβ1 levels. It indicates, that IGF1 stimulates the production of HGF, but not TGFβ1, by HSCs [73]. IGF1 plays role in supporting proliferation of hepatocytes and accelerating DNA synthesis [74], together with IL-6, TNF-α, HGF and TGF-α/EGF systems [75,76]. It was also investigated, using an in vitro, HSC-T6 cell model, that IGFBPrP1 induces liver fibrosis, by mediating the activation of HSCs, accompanied by hepatocyte apoptosis in a Smad3-dependent mechanism [56]. 

All that data suggests the important role of the IGF system components for supporting proliferation of hepatocytes and initiation of HSC proliferation during fibrogenesis [71,72]. It has been described that the components of IGF system stimulate liver regeneration and tissue repair. However, it is still unclear why IGF1 stimulates liver regeneration more effectively than the growth of an intact liver [35,77,78,79]. 

### 4.3. Role in Hepatocellular Carcinoma (HCC)

In in vitro conditions, it has been shown that both IGFs and IGF1R are potent mitogens that contribute to liver carcinogenesis [80,81]. IGF1 exerts a mitogenic effect, mainly through stimulation of DNA synthesis and cyclin D1 expression [7]. 

Recently, it was shown, that IGF1 has a positive effect on HCC growth and metastasis, through inhibition of proteasome-mediated cathepsin B (CTSB) degradation. In HCC cell lines (Hepa1-6 and H22), IGF1 did not change the CTSB mRNA levels, but prolonged the half-life of cathepsin B [82]. In different human HCC lines, high IGF2 levels were found [80,83,84,85]. IGF2 overexpression was connected with HCC hypervascularization [83,84]. Inhibition of IGF2 mRNA and protein levels was associated with decreased cell proliferative activity in these cell lines [80,83,86]. Overexpression of IGF2 in human HCC cells, results from the reactivation of the fetal promoter (P2–P4) pattern, downregulation or deletion of IGF2R, and/or downregulation of IGFBPs. Administration of IGF1R-selective inhibitors (A12) reduced IGF1-induced effects and was associated with a significant reduction of HCC tumor growth [87]. 

IGF1R and its substrates, IRS1 and IRS2, were overexpressed in five human hepatoma cell lines (HepG2, Hep3B, HuH7, HuH6, and PLC/PRF5) [88]. Levels of activated IGF1R form (phosphorylated IGF1R protein) were elevated in human HCC cell lines in comparison with normal human hepatocytes [89]. Activation of IGF1R signaling pathway promotes proliferation, survival and migration of the hepatoma cells. Because of its overexpression, IGF1R can become a potential target for HCC treatment. Inhibition of IGF1R by NVP-AEW541, or AVE 1642 (anti-IGF1R antibodies), results in cell degradation, growth inhibition and cell cycle arrest in HCC cell lines: SK-Hep-1, Hep-3B, Hep-G2 and Huh-7 [88,90]. 

The involvement of IGFBPs in liver carcinogenesis was also observed. IGFBP1 participates in cellular invasion process of human hepatoma cells lines. The expression of IGFBP1 decreases gradually in HCC cell lines (HuH-7, HepG2, SMMC-7721, MHCC97-H). Treatment with IGFBP1 was connected with a significant decrease in the number of invasive cells in HepG2 and MHCC97-H lines [91]. IGFBP1 gene expression has *also* became undetectable in rapidly proliferating hepatoma cells [92]. 

It was also discovered, by in vitro studies, that the dominating pathways, activated by IGF1 in hepatocytes and hepatoma cell lines, involve the PI3K/Akt, as well as signal transducer and activator protein family (STAT), signalling pathways [93,94,95].

In summary, in vitro studies demonstrated that IGF system components contribute to HCC development. Using this model, several impressive mechanisms of the IGF system activity were revealed.

## 5. Evidence from Epidemiologic and Clinicopathological Studies

### 5.1. Role in Nonalcoholic Fatty Liver Disease (NAFLD)

The prevalence of NAFLD, including the more aggressive non-alcoholic steatohepatitis (NASH), is increasing with the growing epidemics of diabetes and obesity. NASH can progress to cirrhosis and its related complications [26,29]. The epidemiologic studies supported previous findings of experimental research, concluding that the IGF1 system may be involved in the pathogenesis of NAFLD/NASH [96].

Serum IGF1 levels in NAFLD patients were significantly lower than in the controls [96,97,98,99]. Arturi et al. showed reduced IGF1 levels in NAFLD patients and suggested, that hepatic insulin resistance may affect IGF1 levels by modulating GH-stimulated synthesis of hepatic IGF1 [97]. Similar results were observed in children and adolescents [100,101]. Several studies suggest an association between serum IGF1 levels and advanced fibrosis in NAFLD patients [37,98,102,103,104]. IGF2 was also associated with fibrosis in NAFLD [105]. IGF1 was the major component of NAFLD activity score in children [105] and adults [32]. Age-standardized IGF1 level was negatively associated with advanced fibrosis [98,104,106] and lobular inflammation [98,103]. IGF1 levels were lower in steatosis with normal (SNLFT) and disturbed liver function tests (SDLFT) in humans with NAFLD [96]. Significant depletion of IGF1 was also observed, after adjustment for age, BMI, and diabetes diagnosis [97,103]. 

Associations between IGF1 serum levels and steatosis, in NAFLD/NASH patients, are also presented in literature, but there are some differences in the presented data. Dichtel et al. showed, that serum IGF1 levels were lower in higher fibrosis stage and patients with NASH, than those without NASH. However, steatosis was not significantly associated with serum IGF1 levels by any measure [103]. Sumida et al. pointed at significantly lower levels of IGF1 in NASH, compared with NAFLD patients [98]. In Spanish patients, IGF1 levels decreased along with the progression of NASH [107]. Argentinian studies also showed that IGF1 levels decreased with progression of liver steatosis in NAFLD patients [108]. Others confirmed that IGF1 levels were negatively associated with hepatic steatosis [109].

NAFLD patients with advanced fibrosis had higher levels of IGFBP1 [104]. Fasting serum phosphorylated IGFBP1 (fS-pIGFBP1) can be used as one of the top noninvasive predictors of liver fat in NAFLD [110]. Serum IGFBP5 levels were also correlated with fibrosis and NASH scores in NAFLD [102]. IGFBP3 levels were higher in NAFLD patients [109], as well as in cases of advanced steatosis in biopsy-confirmed NAFLD [106]. IGF1/IGFBP3 ratio is used as an index of the bioavailability of IGF in the circulation. In studies by Chischima et al., the IGF1/IGFBP3 ratio was not correlated with histological features of NAFLD [106]. Different results were presented by other authors. IGF1/IGFBP3 ratio revealed a tendency to decrease in patients with steatosis, confirmed by ultrasound and increased aminotransferases [111], as well as the ones with NAFLD and portal fibrosis [37]. Moreover, the correlation between IGF1/IGFBP3 and severity of NAFLD retains significance, after adjustment for age, gender, race/ethnicity, homeostasis model assessment for insulin resistance (HOMA-IR) and adiposity [112]. Observations regarding NAFLD in children seem to be interesting, with IGF1/IGFBP3 ratio recognized as the major predictor of liver inflammation [105]. Recently published data reveals, that miR-190b inhibition suppressed lipid accumulation and improved insulin sensitivity by targeting IGF1 and ADAMTS9, suggesting that miR-190b inhibition may be a therapeutic strategy against NAFLD [113].

Summarizing, the epidemiologic data on IGF system and NAFLD/NASH showed that low serum IGF1 levels, together with higher IGFBP levels (IGFBP1 and -3, -5) are associated with increased severity of the NAFLD. However, the role of IGF1/IGFBP3 ratio as a predictor of impaired liver function in NAFLD/NASH remains to be better characterized. That data suggests that evaluation of circulating IGF1, together with the proinflammatory markers, might be useful to assess the severity of the NAFLD and can be target for novel form of NAFLD therapeutic strategy. 

### 5.2. Role in Liver Cirrhosis

In each etiology of liver cirrhosis, similar IGF system disturbances are observed, namely GH resistance and IGF1 deficiency, with a variety of resulting metabolic complications [34]. In developed liver cirrhosis, decreased concentrations of IGF1 were observed, in comparison with healthy individuals [114,115,116,117,118,119,120,121,122]. Similar changes in IGF1 concentrations, depending on the clinical stage of liver cirrhosis, are also described in children [123]. IGF1 concentration decreased with the severity of cirrhosis (Child–Pugh score), reaching significantly low values in class C [115,116,124,125,126]. In coinfected HCV/HIV patients, long term reduction of IGF1 levels was also significantly associated with liver stiffness, evaluated in elastography, regardless of HIV status and age [127]. The negative correlation between IGF1 and MELD was found [125,126,128]. Correlations of serum IGF1 levels with other markers of liver function, including positive correlation with albumin [116,125], and negative with INR, aPTT ratio [125] and spleen size [116], were also found. IGF1 values of <30 ng/mL were a negative prognostic factor in patients with liver cirrhosis. Patients with such low IGF1 values died within six months [115]. In decompensated liver cirrhosis, IGF1 levels are independently related to mortality. The Kaplan–Meier analysis showed a 90-day survival probability of 94.3%, in patients with IGF1 ≥13 ng/mL and 63.2% for cases with IGF1 <13 ng/mL [129]. Longitudinal observation of liver cirrhotic patients showed a decrease in IGF1 concentration in those who developed primary liver cancer. This reduction occurred about 9 months before HCC development [130]. 

IGF1 can also play the role of a marker of liver function after liver transplantation. In cases with IGF1 levels persistently lower than 90 mUI/mL, later than one week from surgical procedure, short-term survival time was observed in less than 13% [131]. A rapid and significant increase in IGF1 concentrations were noted in patients after liver transplantation due to cirrhosis [132]. In patients with cirrhosis, there is a significant reduction in serum IGF2 levels, in relation to healthy people. Also, similarly to IGF1 levels, differences in concentrations between clinical categories of cirrhosis (Child Pugh score) were observed [115,133]. Very low IGF2 values (<200 ng/mL) were poor prognostic factors, with patients dying within six months [115]. After liver transplantation, a significant increase in IGF2 levels was noted in almost all patients [132]. 

Similar observations, such as those of IGF1 and IGF2, were done for IGFBP3 levels, which were significantly lower in cirrhotic patients than in controls [118,121,125,128], in children [123], as well as in comparison with people with chronic hepatitis C [117,118]. IGFBP3 serum levels, such as IGF1, were related to severity of liver cirrhosis. Negative correlations with Child–Pugh and MELD scores, creatinine, INR, total bilirubin and aPTT ratio were present [121,125,128,134]. There was no significant difference in IGFBP3 concentrations in patients with liver cirrhosis depending on its etiology (alcohol vs. HBV vs. HCV) [116]. At the six-month follow-up, cirrhotic patients with IGFBP3 levels lower than 6 ng/mL died from hepatic failure and/or bleeding during this period [115]. IGFBP3 levels show significant negative correlation with α-fetoprotein (AFP) levels, with its cut-off value of <682.6 ng/mL discriminating between liver cirrhosis and HCC [121]. After liver transplantation, both IGF1 and IGFBP3 serum levels normalization was observed [128,132,134]. In alcoholic cirrhosis, serum IGFBP1 and IGFBP2 levels were elevated when compared with controls [119]. 

The studies at the tissue level of fibrotic and cirrhotic human liver specimens generally confirm the epidemiologic data. Donaghy et al. noted significantly decreased hepatic IGF1 and ALS mRNA (in parallel with serum proteins), as well as unchanged hepatic IGFBP3 mRNA expression [135]. Compared with noncirrhotic liver, all cirrhotic specimens in other studies showed reduced hepatocellular expression of M6P/IGF2R protein, which contrasted with enhanced expression in perisinusoidal cells [136]. The studies on IGF2 in liver cirrhosis indicate that IGF2 plays a role in cell proliferation of regenerating nodules, as well as in the development of HCC [136,137]. High focal expression of IGF2 RNA was found in some hepatocytes of all livers with HBV- or HCV-induced cirrhosis, but in only one of the cirrhoses with a nonviral etiology [136]. Some other studies also suggested that, in liver cirrhosis and in some benign liver tumors, premalignant proliferative states might be identified by the presence of IGF2 fetal transcripts [138]. In the case of IGF1R, overexpression in hepatocytes has been described in liver cirrhosis, when compared with normal livers. Additionally to increased expression of IGF1R, increased levels of insulin receptor, IRS2, and MAPK were observed in the glycogen storing foci of the altered hepatocytes [63]. Similarly, IGFBPrP1 (IGFBP7) have been shown to be significantly increased in expression in human fibrotic and cirrhotic liver specimens, with positive correlation between the expression and the number of collagen fibers in HSCs observed. siRNA-mediated gene silencing of IGFBPrP1, resulted in significantly decreased levels of collagen I and fibronectin in HSCs [139].

In summary, the epidemiologic data and research performed at the tissue level documented the changes in nearly all IGF system components in liver cirrhosis. Several of them could be possible markers of hepatocellular dysfunction, or of the functional reserve of hepatocellular functional capacity, malnutrition and survival. Some of studies suggest that monitoring of the IGF system before and after liver transplantation could be of value in the investigation of the transplantation outcome. On the other hand, elevated levels of tissue expression of IGF2, IGF1R or other IGF system components (IGFBP7), may indicate an increased risk of hepatocellular carcinoma. 

### 5.3. Role in Hepatocellular Carcinoma (HCC)

Several direct or indirect epidemiologic lines of evidence add further support to an association of the IGF system with the development of HCC. IGF1 seems to play an important role in the development and progression of CLD and the development of HCC.

In cases of HCC, significant reduction of serum IGF1 levels as compared to cirrhosis [121,133,140,141] and healthy controls [133,140,142] was observed. The reduction in IGF1 was greater for the virus-associated HCC than in noninfected HCC patients [143]. Similarly, Su et al. showed lower IGF1 levels in HCC patients with HCV and/or HBV than those not infected, as well as lower levels of IGF1 in the HCV+ group than in the HBV+ group [144]. It was shown that serum IGF1 levels predict prognosis in cases of HCC. Lower plasma IGF1 levels were significantly correlated with advanced clinical parameters, shorter time to progression and poor overall survival [140,141,145,146,147,148,149], even in patients that underwent transarterial chemoembolization (TACE) [148,149,150,151,152] or other types of curative treatment, such as surgical resection or percutaneous ethanol injection [150]. Longitudinal observation of HCC cases (median follow-up period of 41.8 months) proved that IGF1 level was an independent predictor of poorer survival [151]. Also, in patients undergoing liver resection for HCC, preoperative low and delayed recovery of IGF1 levels at 30 days after surgery were independent risk factors for early recurrence of this cancer [153]. 

Serum IGF2 levels were significantly higher in HCC than in liver cirrhotic patients, but lower than in controls [121,140,154,155,156]. Strong correlation between IGF2 and AFP levels and tumor diameter was found [156]. The circulating IGF2 mRNA was present in one third of HCC. It was correlated with the stage of HCC (all HCC with extrahepatic metastasis and ~one third HCC with low AFP levels) [154]. The multivariate analysis showed, that either IGF2 or AFP are associated with an increased risk of HCC presence [155]. 

In HCC patients, the IGFBP3 concentration was significantly lower than in cirrhotic ones [121,133,141,157]. This diminution is similar to cases of poor nutrition, disturbed hepatic function, and decreased GH secretion [158]. After controlling for some data (hepatitis infection, BMI, smoking and alcohol abuse), a higher molar difference of IGFBP3 and IGF1 was connected with a decreased hepatoma risk, more than IGFBP3 alone [159]. There are different data about prognostic role of serum IGF1/IGFBP3 ratio in HCC. The increased rate of serum IGF1/IGFBP3 is described in patients with HCC, compared to patients with cirrhosis and liver failure [157]. On the contrary, the IGF1/IGFBP3 ratio decreased in patients with liver cancer [142]. Recent data do not indicate that IGF1/IGFBP3 is associated with an increased risk of HCC development [141,159]. Compared to healthy individuals, in patients with primary liver cancer, a higher concentration of IGFBP1 was observed [142]. The elevated IGFBP2 serum levels seem to be indicators of tumor activity. In HCC patients, these levels were markedly high [158]. IGFBP7 binds the IGF1R and functions as a candidate tumor suppressor. IGFBP7 promoter methylation in HBV-associated HCC, was significantly higher than in chronic hepatitis B and healthy control. Elevated IGFBP7 methylation frequency was significantly higher in HCC with vascular invasion than without this complication [160]. 

The studies on tissue mRNA and protein levels of human HCC specimens generally confirm the epidemiologic data. All tested HCC tissues showed a decrease in IGF1 expression (mRNA, protein) [161,162,163]. Significant correlations between reduced IGF1 expression in primary lesions and poor differentiated HCC and portal vein infiltration were also found [163]. It is interesting that survival time in HCC patients treated with resection of the tumor is closely related with IGF1 expression in the liver tissue adjacent to tumor. Higher IGF1 expression was connected with shorter survival when compared to equal or lower expression (22 months vs. 72 months, respectively) [164]. IGF2 expression was increased in tumor and adjacent background, nontumor tissue [164]. IGF2 mRNA expression was present in all HCC tissues, and was absent in noncancerous tissues [154]. 40- to 100-fold increase in IGF2 mRNA expression was observed in HCC, as compared to normal liver [138]. Similarly to IGF1, expression of IGFBP3 (both at the mRNA and protein levels) was either undetectable or low in HCC, when compared with adjacent normal tissues [161]. On the contrary, in some studies, IGF1 mRNA expression was higher—but IGFBP3 mRNA expression was lower—when tumor tissues were compared to that of adjacent nontumor tissue [165]. 

The downregulation in expression of IGFBP1, -3 and -4 mRNA in HCC tissues was observed when compared to cirrhotic and normal liver tissues [91,166]. Low levels of IGFBP3 expression in HCCs were correlated with tumor size, tumor multiplicity, node, metastasis, clinical stage and shorter survival time [167]. Positive correlations between decreased expression of IGFBP1 and tumor differentiation, liver cirrhosis, microvascular invasion or metastasis, TNM stage and poor survival were found. The study concluded that low levels of IGFBP1 could play role of an independent prognostic factor for the survival of patients with HCC [91]. Moreover, IGFBP7 expression was significantly downregulated in HCCs, compared to normal liver. Lower IGFBP7 expression correlated with poor postoperative prognosis and inversely with the stages and grades of HCC [168,169]. 

Studies performed on IGF1R tissue expression, showed that tumor expression was significantly higher than in the surrounding tissue and correlated with the HCC differentiation and cirrhosis, but not to the number or size of tumors, HBV infection, and AFP level [170,171]. On the contrary, there is data showing low IGF1R expression in HCC tumor samples [161,164]. IGF1R expression in adjacent noncancerous tissue was connected with underlying disease (high expression in alcoholic hepatitis) [164]. 

Currently, the innovative molecular techniques allowing us to study the alternative splicing of the IGF family genes result in multiple isoforms, which give rise to different peptides—their role still not completely known. The literature data confirms that some of the C-terminal E-peptides of IGF1 can modulate its actions, stability, or bioavailability, or possess other surprising properties [14,15,172].

In summary, decreased serum concentration of IGF1 seems to be a potential risk factor for HCC progression, while levels of circulating IGF2 (increased or decreased in HCC, according the different data) is a rather uncertain marker of hepatocarcinogenesis. Clinicopathological data, involving tissue expression of IGF system components in patients with HCC, confirmed that the decreased IGF1 secretion, an increased tissue expression of IGF1R, accompanied by a reduced expression of IGFBPs (mainly of IGFBP3), could also be used as prognostic markers in HCC, independent prognostic factors of HCC survival (IGFBP1), or may function as a tumor suppressor (IGFBP7). 

However, questions still remain. Is decreased synthesis of IGFs system components dependent on chronic damage of liver parenchyma, or is damaged parenchyma an effect of an altered function of GH/IGF/IGFR system? 

The most common observations on serum levels and tissue expression changes in the IGF/IGFR pathway in chronic liver diseases are summarized in Table 1.

## 6. Genomic Alterations of IGF System in CLD 

The published results only contain data regarding some genomic alterations in IGF2 and IGF2R genes. Hypomethylation at the IGF2 locus may be predictive for HCC in cases of liver cirrhosis and hepatitis C infection [173,174]. The presence of IGF2+3580 AA genotype, IGF2+3123 GG genotype, or G allele, were significantly connected with HCC risk. Moreover, combination of IGF2+3580 AA homozygosity and IGF2R 1619 GG homozygosity, showed low risk for HCC and presented a significant protective effect against HCC [175,176]. The T allele (TT+CT genotype) at position −13021C in IGF2 was independently connected with HCC recurrence after curative surgical resection [177]. 

## 7. IGF System in CLD—Summary and Outstanding Questions

**Figure 1:**
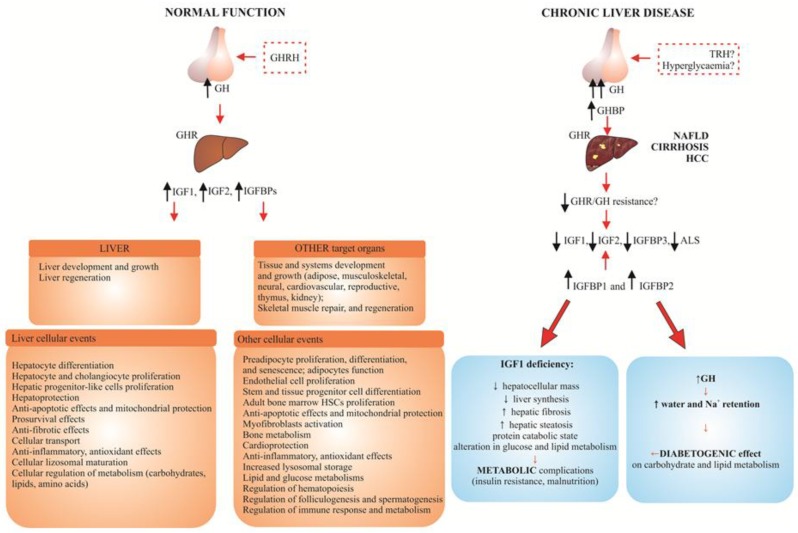
Schematic presentation of the GH-IGF system in normal human liver function and in the most common chronic liver disease (NAFLD, liver cirrhosis and HCC) [25,34,35].

As presented in Figure 1, in patients with CLD, strongly elevated GH plasma levels and decreased hepatic responsiveness to GH are observed, which result in low IGF1, IGF2, IGFBP3 and ALS plasma levels. These changes result in well-known, clinical symptoms of IGF1 deficiency [25,34,35].

The main findings of the review and conclusions:

1. The studies on the IGF1R signaling pathway in CLD using animal models and in vitro studies, allowed for the clarification of general aspects of hepatocarcinogenesis (cell cycle progression, uncontrolled proliferation, cell survival, migration, inhibition of apoptosis, protein synthesis and cell growth), as well as show that systemic IGF1 administration can reduce fibrosis and ameliorate general liver function. This allowed them to become promising factors in development of new methods of managing the most common chronic liver diseases.
2. The results of several careful epidemiologic studies concerning CLD show that low IGF1 serum level is a typical indicator of decreased hepatic reserve in NAFLD, liver cirrhosis and HCC (Table 1, Figure 1). Different values of lowered serum concentration of both IGFs were proposed as a negative prognostic factor in patients with liver cirrhosis (IGF1: 13, 30 ng/mL; IGF2: below 200 ng/mL). In the majority of HCC patients, the inverse association between serum IGFBP3 level and the risk of cancer was observed.
3. Clinicopathological data involving tissue expression of IGF system components in patients with CLD confirmed that decreased IGF1 secretion and increased expression of IGF1R, accompanied by a reduced expression of IGFBPs (mainly of IGFBP3), as compared to the control, are negative prognostic markers in CLD, independent prognostic factors of survival (IGFBP1), or may function as a tumor suppressor (IGFBP7) in HCC.
4. The main outstanding question still remains—is the decreased synthesis of the IGF system components due to chronic damage of liver parenchyma, or is damaged parenchyma an effect of an altered function of GH/IGF/IGFR system?
5. Promising results of the studies on IGF system are coming from genetic and epigenetic investigations, as well as from research on the role of various isoforms of IGF system components in hepatocarcinogenesis, and cooperation between functioning IGF1R and many viral and cellular oncogenes.
6. Concluding the article, we believe that addressing some of those questions will be essential to fully understand the mechanisms of IGF system signaling in chronic liver diseases and to develop new IGF-based biomarkers and treatment strategies for liver cancer.

## Figures and Tables

**Table 1:** Serum levels and tissue expression changes in the IGF/IGFR pathway in chronic liver diseases.

Gene/Protein		NAFLD	Liver Cirrhosis	HCC
IGF1	S	↓[97,100,101] ♣[37,98,102,103,104,106]	↓[114,115,116,117,118,119,120,121,122] ♦[115,116,124,125,126,128]	↓[133,140,142] ⇓[121,133,140,141] #[140,141,145,146,147,148,149,150,151,152]
	T	↓[65] ♣[32]	nd	↓[161,162,163]
IGF2	S	♣[105]	↓[115] ♦[115,133]	⇑, ↓[121,140,154,155,156]
	T	nd	↑[136]	↑[138,154,164]
IGF1R	S	nd	nd	nd
	T	nd	nd	↓[161,164] ↑[154,171]
IGFBP1	S	↑[104]	↑[119]	↑[142]
	T	nd	nd	↓⇓[166] #[91]
IGFBP2	S	nd	↑[119]	↑[158]
	T	nd	nd	nd
IGFBP3	S	↑[106,109]	↓[118,121,123,125,128]	⇓[121,133,141,157]
	T	↓[65]	nd	↓[161,165,166] ⇓[166]
IGFBP4	S	nd	nd	nd
	T	nd	nd	↓⇓[166]
IGFBP5	S	♣[102]	nd	nd
	T	nd	nd	nd
IGFBP7	S	nd	nd	↑[160]
	T	nd	↓[139]	↓ #[168,169]

Legend: S—serum concentration; T—tissue level; ↑/↓—significant increase/decrease, as related to control individuals; ♣—association between IGF1 levels with advanced fibrosis; ♦—association between IGF1/IGF2/IGFBP3 levels with clinical stage of cirrhosis; ⇓/⇑—significant decrease/increase as related to cirrhosis; #—significant correlation with advanced clinical parameters, shorter time to progression, and poor overall survival; nd—no data; no. of ref.—numbers of references in order of citation (for details, see text).

## Abbreviations

AA	adenine-adenine
ADAMTS9	a disintegrin and metalloproteinase with thrombospondin motifs 9
AFP	alpha-fetoprotein
Akt (AKT) (protein kinase B)	serine/threonine protein kinase
ALD	alcoholic liver disease
ALS	acid-labile protein subunit
aPTT	Activated Partial Thromboplastin Time
BMI	body mass index
CLD	chronic liver diseases
CT	cytosine-thymine
ECM	extracellular matrix
EGF/R	epidermal growth factor/receptor
ERK	extracellular signal-regulated kinase
GG	guanine-guanine
GH	growth hormone
HBV/HCV	hepatitis B/C virus
HCC	hepatocellular carcinoma
HGF	hepatocyte growth factor
HIV	human immunodeficiency virus
HSCs	human stellate cells
IGF	insulin-like growth factor
IGFR	IGF receptor
IGFBPs	IGF binding proteins
IGFBPrP1-10	IGF IGFBP-related proteins 1-10
IL	interleukin
INR	International Normalized Ratio of prothrombin time of blood coagulation
IR	insulin receptor
IRS-1/2	insulin receptor substrate 1/2
kDa	kilodalton
LH	luteinizing hormone
MAPK	mitogen-activated protein kinase (originally called ERK)
MELD	Model of End-Stage Liver Disease
NAFLD	nonalcoholic fatty liver disease
NASH	nonalcoholic steatohepatitis
PI3K	phosphatidylinositol 3-kinase
STAT	signal transducer and activator of transcription
TGF-α, -β	transforming growth factor α, -β
TNF-α	tumor necrosis factor α
TNM	classification of Malignant Tumors (T—tumor; N—lymph nodes; M—metastasis)
TT	thymine-thymine
